# EMT-Related Genes Have No Prognostic Relevance in Metastatic Colorectal Cancer as Opposed to Stage II/III: Analysis of the Randomised, Phase III Trial FIRE-3 (AIO KRK 0306; FIRE-3)

**DOI:** 10.3390/cancers14225596

**Published:** 2022-11-14

**Authors:** Elise Pretzsch, Volker Heinemann, Sebastian Stintzing, Andreas Bender, Shuo Chen, Julian Walter Holch, Felix Oliver Hofmann, Haoyu Ren, Florian Bösch, Helmut Küchenhoff, Jens Werner, Martin Konrad Angele

**Affiliations:** 1 Department of General, Visceral, and Transplant Surgery, Ludwig-Maximilians-University Munich, 80539 Munich, Germany; 2 German Cancer Consortium (DKTK), German Cancer Research Center (DKFZ), 69120 Heidelberg, partner site 80336 Munich, Germany; 3 Department of Hematology/Oncology and Comprehensive Cancer Center Munich, LMU University Hospital Munich, Ludwig-Maximilians-University Munich, 81377 Munich, Germany; 4 Comprehensive Cancer Center (CCC Munich LMU), LMU University Hospital Munich, 81377 Munich, Germany; 5 Department of Hematology, Oncology, and Cancer Immunology (CCM), Charité-Universitätsmedizin Berlin, Freie Universität Berlin, Humboldt-Universität zu Berlin, Berlin Institute of Health, 10117 Berlin, Germany; 6 Statistical Consulting Unit, StaBLab, Department of Statistics, LMU Munich, 80539 Munich, Germany; 7 Munich Center for Machine Learning, LMU Munich, 81377 Munich, Germany; 8 Department of General, Visceral, and Pediatric Surgery, University Medical Center Goettingen, 37075 Göttingen, Germany

**Keywords:** colorectal cancer, metastasis, EMT, EMT-related genes

## Abstract

**Simple Summary:**

Despite huge advances in local and systemic therapies, the 5-year relative survival rate for patients with metastatic CRC is still low. To avoid over- or undertreatment, proper risk stratification with regard to treatment strategy is highly needed. As EMT (epithelial-mesenchymal transition) is a major step in metastatic spread, this study analysed the prognostic effect of EMT-related genes in stage IV colorectal cancer patients using the study cohort of the FIRE-3 trial, an open-label multi-centre randomised controlled phase III trial of stage IV colorectal cancer patients. Overall, the prognostic relevance of EMT-related genes seems stage-dependent. EMT-related genes have no prognostic relevance in stage IV CRC as opposed to stage II/III.

**Abstract:**

Introduction: There is no standard treatment after resection of colorectal liver metastases and the role of systemic therapy remains controversial. To avoid over- or undertreatment, proper risk stratification with regard to postoperative treatment strategy is highly needed. We recently demonstrated the prognostic relevance of EMT-related (epithelial-mesenchymal transition) genes in stage II/III CRC. As EMT is a major step in CRC progression, we now aimed to analyse the prognostic relevance of EMT-related genes in stage IV CRC using the study cohort of the FIRE-3 trial, an open-label multi-centre randomised controlled phase III trial of patients with metastatic CRC. Methods: Overall and progression free survival were considered as endpoints (*n* = 350). To investigate the prognostic relevance of EMT-related genes on either endpoint, we compared predictive performance of different models using clinical data only to models using gene data in addition to clinical data, expecting better predictive performance if EMT-related genes have prognostic value. In addition to baseline models (Kaplan Meier (KM), (regularised) Cox), Random Survival Forest (RSF), and gradient boosted trees (GBT) were fit to the data. Repeated, nested five-fold cross-validation was used for hyperparameter optimisation and performance evaluation. Predictive performance was measured by the integrated Brier score (IBS). Results: The baseline KM model showed the best performance (OS: 0.250, PFS: 0.251). None of the other models were able to outperform the KM when using clinical data only according to the IBS scores (OS: 0.253 (Cox), 0.256 (RSF), 0.284 (GBT); PFS: 0.254 (Cox), 0.256 (RSF), 0.276 (GBT)). When adding gene data, performance of GBT improved slightly (OS: 0.262 vs. 0.284; PFS: 0.268 vs. 0.276), however, none of the models performed better than the KM baseline. Conclusion: Overall, the results suggest that the prognostic relevance of EMT-related genes may be stage-dependent and that EMT-related genes have no prognostic relevance in stage IV CRC.

## 1. Introduction

Colorectal cancer (CRC) is the third most common cancer worldwide, being the second leading cause of cancer-related deaths [1] with more than 1.9 million new cases each year. About 20% of patients present with synchronous liver metastases and up to 50% of patients develop distant metastases during their disease with the liver being the most frequent site of metachronous spread [1,2,3,4,5]. Despite huge advances in local and systemic therapies, the 5-year relative survival rate for patients with metastatic CRC (mCRC) still ranges between 14–17% [6]. To date, the personalised approach to treat mCRC as recommended by national (German S3-Leitlinie, NCCN) and international (ESMO, ESMO-Asia) guidelines is limited to the analysis of microsatellite (MSI) status and mutational analysis of RAS (rat sarcoma oncogene) and B-RAF [7,8,9,10]. However, extended molecular testing has the potential to identify druggable targets beyond standardised treatment options, establish biomarkers that allow better and more precisely risk stratification, predict prognoses, and improve clinical decision-making in a precision medicine approach. There is still no standard treatment after the resection of colorectal liver metastases (CRLM) and the role of systemic therapy remains controversial. Whereas adjuvant treatment is recommended after surgery in stage III CRC with nodal spread, there is no standard recommendation for systemic treatment after surgery in stage IV CRC with distant spread, a rationale that might not seem conclusive [9]. To avoid over- or undertreatment, a proper risk stratification regarding the postoperative treatment strategy is highly needed (precision oncology). Successful stratification of risk groups based on tumour biology reflected in longer disease-free survival in high-risk groups receiving additive systemic treatment and avoidance of unnecessary adverse effects of systemic treatment in low-risk groups could lead to a paradigm shift in the treatment strategy of stage IV CRC.

In this respect, we recently demonstrated the prognostic relevance of EMT-related (epithelial-mesenchymal transition) genes in stage II/III CRC. Further, we proposed an EMT-related gene signature that identified patients at risk of relapse in multiple CRC cohorts. This EMT-related gene signature was a strong predictive indicator for recurrence in stage II/III CRC patients and associated with overall survival [11]. With the aim to optimise patient outcome, the respective EMT signature might help to stratify patients according to their tumour biology, and contribute to personalised treatment in the future.

EMT is a key program that enables stationary epithelial cells to lose their cell-cell adherence and acquire mesenchymal properties, including enhanced mobility, invasiveness, increased resistance to apoptosis, and degradation and production of extracellular matrix components, that are all essential for invasion and metastasis. In this respect, EMT is associated with an aggressive phenotype, pivotal for tumour progression and the prerequisite for metastatic spread [12,13]. EMT is regulated at different molecular levels that lead to the loss of E-Cadherin as the critical event with a subsequent activation of all major cancer cell intrinsic signaling pathways. Whereas EMT and EMT-related gene signatures have been demonstrated to be associated with prognosis and therapeutic resistance in non-metastatic stages of CRC and various other tumour entities [11,14,15,16,17], the prognostic role of EMT-related genes and our previously proposed EMT-related gene signature in mCRC remains uncertain.

As EMT is a major step in CRC progression and metastatic spread, it was the aim of this study to analyse the prognostic relevance of EMT-related genes in stage IV CRC patients using the study cohort of the FIRE-3 trial. The FIRE-3 trial was an open-label multi-centre randomised controlled phase III trial for first-line treatment of patients with RAS wild-type (wt) mCRC patients [18]. In this respect, we aimed to assess whether the prognostic value of the previously identified EMT-related genes and our proposed EMT-related gene signature in stage II/III CRC can be validated in the metastatic setting of CRC and potentially be used for risk stratification and guidance of systemic treatment in an individualised therapy approach in stage IV CRC.

## 2. Materials and Methods

### 2.1. Study Design

FIRE-3 was designed as an open-label, multi-centre, randomised phase III trial that evaluated the combination of FOLFIRI plus cetuximab or bevacizumab as first-line regimen in irresectable RASwt mCRC. Treatment protocol, regulatory aspects of trial conduct, safety and efficacy, outcome, molecular subgroups, and next-generation sequencing results were published in the studies by Heinemann et al., Stintzing et al., Stahler et al., and Modest et al. [14,18,19,20,21,22].

### 2.2. Patients

Clinical data was available from 752 patients. In 416 cases, genetic information was also available. After removing missing values and a single subject with primary tumour location on both sides (rather than left or right), 350 patients with complete data remained for the analysis, of which 237 were male and 113 were female. For each patient, the corresponding treatment, tumour location, metastatic status (solitary liver metastasis (nonresectable metastases confined to the liver at time of diagnosis) versus distant metastasis in more than one organ), BRAF^V600E^ status (wild-type (wt) versus mutated (mut)), survival parameters (overall survival (OS), progression-free survival (PFS)) along with 191 variables containing gene expression data were collected (Table 1).

### 2.3. Gene-Expression Analysis

Using formalin-fixed paraffin-embedded (FFPE) samples of primary tumour tissue, gene expression analysis was carried out using ALMAC’s Xcel^TM^ gene- expression array at ALMACs laboratories [23]. All analyses were approved by the ethics committee of the Ludwig-Maximilians-University, Munich (#186-15).

### 2.4. EMT-Related Dataset

The EMT-related dataset investigated in this study was derived from public databases as previously described [11]. In this respect, transcriptome profiles and clinical information of 1780 stage II/III CRC patients from 15 public datasets were investigated. Coefficient variant analysis was used to select reference genes for normalising gene expression levels. Univariate, LASSO, and multivariate Cox regression analyses were combined to develop the originally studied EMT related dataset [11].

### 2.5. Outcomes

Endpoints investigated in this study included progression-free survival (PFS) (time from randomisation to disease progression or death from any cause) and overall survival (OS) (time from randomisation to death from any cause).

### 2.6. Statistical Analysis

For each endpoint (OS and PFS), we trained different models and investigated their predictive performance. In order to investigate the prognostic relevance of gene data, we compared the predictive performance of the models using clinical data only to model using gene data in addition to clinical data, expecting better predictive performance if gene data has prognostic value. The models used for comparison were Kaplan-Meier (KM) [24], (regularised) Cox Regression (Cox) [25], Random Survival Forest (RSF) [26,27], and Gradient Boosted Trees with Cox Loss (GBT) [28]. KM served as a baseline for the prediction without taking any covariate information into account, the Cox models served as a baseline for a model with covariates but without non-linear effects and interactions. The predictive performance of the models was measured by the integrated Brier Score (IBS) evaluated at the median survival time [29].

For regularised Cox, RSF, and GBT, hyperparameter optimisation (HPO) was performed via random search [30] and three-fold cross validation (CV) on the respective training data (see Appendix A for details). Performance evaluation was based on a repeated five-fold CV with five repetitions. All analyses including training the models were performed in the R programming environment (version 4.1.3). For the setup of survival tasks, model training, HPO, and performance evaluation, we used mlr3proba [31] with the mlr3 [32] ecosystem. All code used for the analysis is available from GitHub: https://github.com/adibender/EMT-gene-fire3-prognostic-relevance (accessed on 1 January 2022).

## 3. Results

**Figure 1:**
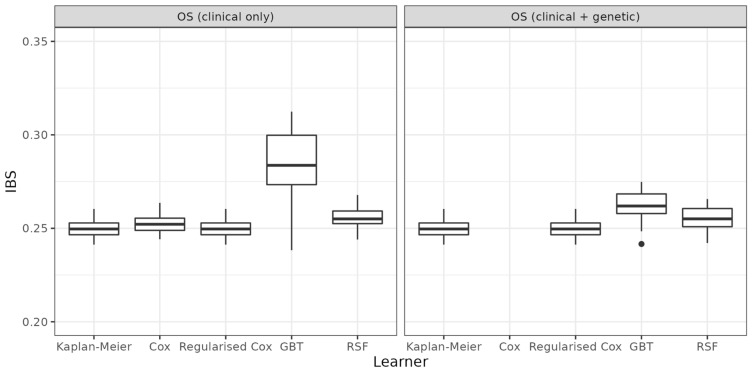
Benchmark experiments with respect to overall survival. Predictive performance of five learners based on 25 values of the IBS. Lower values indicate better performance. Performance based on clinical data only (**left**) is compared to performance based on clinical and gene data (**right**). Performance of the unregularised Cox model is omitted for the high-dimensional setting (data with genes).

**Figure 2:**
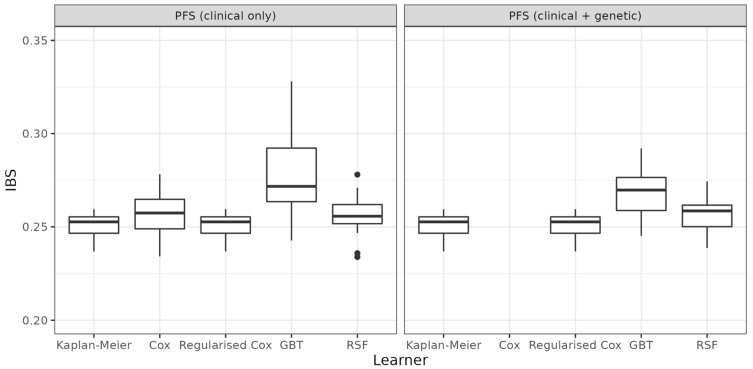
Benchmark experiments with respect to progression-free survival. Predictive performance of five learners based on 25 values of the IBS. Lower values indicate better performance. Performance based on clinical data only (**left**) is compared to performance based on clinical and gene data (**right**). Performance of the unregularised Cox model is omitted for the high-dimensional setting (data with genes).

The results of the benchmark experiments are given in Figure 1 (OS), Figure 2 (PFS), and Table 2. The boxplots indicate the distribution of the 25 IBS values calculated on the test data from the respective iteration of the repeated cross-validation. Lower values of the IBS indicate better predictive performance. The results indicate that predictive performance cannot be improved when using gene data in addition to clinical data (comparison of left and right panels, respectively). While mean values for GBT decrease slightly when comparing performance with and without genetic data (OS: 0.262 vs. 0.284; PFS: 0.268 vs. 0.276), the performance is still worse than the mean performance of RSF or unregularised Cox with clinical data only. Furthermore, none of the models using additional information (clinical and/or genetic) can outperform the KM baseline (OS: 0.250, PFS: 0.251). Performance of regularised Cox regression is identical to KM, as all coefficients are penalised to zero.

## 4. Discussion

Advances in genomic and transcriptomic analyses have shifted cancer therapy towards a precision medicine approach and allowed better understanding of the molecular alterations of CRC with regard to tumour initiation, progression, and resistance [33]. While the TNM staging system in combination with molecular markers (RAS, BRAF, MSI) is the backbone of therapeutic decisions and used as a guideline for survival estimates, there is a wide variation in prognosis among CRC patients with the same TNM stage and a survival paradox of stage II/III CRC patients on account of the inherent heterogeneity that traditional clinicopathological and molecular features fail to explain. In this respect, identification of innovative markers and risk factors based on tumour biology that can guide the administration of systemic treatment (targeted therapies, postoperative additive treatments) in CRC need to be introduced into the clinical arena.

In this respect, we recently demonstrated that EMT-related genes have prognostic relevance in stage II/III CRC. Further, we developed an innovative prognostic model based on our proposed EMT-related gene signature predicting recurrence of stage II/III CRC patients, offering a potential explanation with regard to tumour biology beyond traditional clinicopathological characteristics for the mechanisms underlying the observed survival paradox [11]. We now aimed to assess the prognostic relevance of EMT-related genes in a metastasized CRC setting using the study cohort of the FIRE-3 trial, a multi-centre randomised controlled phase III trial of mCRC patients [18].

Analyses using (regularised) Cox and RSF showed no improvement in predictive performance according to IBS when using gene data in addition to clinical data (see Figure 1 and Figure 2; Table 2), and therefore no prognostic effect of EMT-related genes in stage IV CRC. GBT performed slightly better when using gene data in addition to clinical data, but still performed worse than the RSF or unregularised Cox using clinical data only. Furthermore, according to our results, none of the models using covariate information (clinical and/or gene data) could outperform the KM with respect to predictive performance.

We have previously shown that FOLFIRI plus cetuximab was associated with improved OS in patients with RASwt mCRC relative to those treated with FOLFIRI plus bevacizumab [18]. This FOLFIRI plus cetuximab conferred OS benefit, however, was in the absence of differences in investigator-assessed objective response or PFS. In an attempt to elucidate the underlying relationship for these unexplained results, metrics of tumour dynamics were assessed, and centralised radiological review revealed that FOLFIRI plus cetuximab induced superior objective response, frequency of early tumour shrinkage and depth of response compared with FOLFIRI plus bevacizumab. In this respect, early tumour shrinkage and depth of response were associated with OS in both treatment groups [19]. These results highlight the importance of new innovative metrics that reflect tumour biology to predict therapeutic response and outcome. Indeed, this is underscored by the increasing evidence that evaluation of response according to RECIST criteria may not adequately capture the quality and quantity of response to targeted therapies in mCRC [24]. As depth of response is an on-treatment parameter occurring approximately 3.5 months after the beginning of treatment, this parameter might rather be used for retrospective analyses than initial clinical decision making. On the other hand, early tumour shrinkage is a useful parameter to guide decision making in the early phase of treatment, however, the parameter by itself does not consider the subgroup of patients that show no early shrinkage, though they are slow responders who could still benefit from continuation of treatment. In this respect, there is still a need for further metrics to supplement the RECIST criteria that are easy to obtain, feasible in a clinical setting, and can predict therapeutic efficacy not only with improved precision, but also at an early stage. To this aim, we assessed the relevance of our proposed EMT-related signature that fulfilled the above-described requirements and was able to predict recurrence and survival in stage II/III CRC patients, in a metastatic setting.

To our knowledge, we are first to assess the prognostic relevance of EMT-related genes in stage IV CRC. The results of this study indicate that in an advanced stage, EMT-related characteristics may give insights into the underlying tumour biology and mechanisms that have preceded metastatic spread but do not add further value to the determination of prognosis.

In fact, the role of EMT in metastatic tumours is still under debate. EMT is a critical process for tumour progression in which epithelial cells lose their epithelial features and acquire mesenchymal characteristics, such as invasion and motility. During EMT, cancer cells are considered to gain a more aggressive phenotype and are more prone to develop metastatic spread. Studies suggest that induction of EMT is critical for the initial steps of metastatic spread but not for metastatic seeding and outgrowth at the distant site. Other studies point out that the mere presence of tumour cells displaying EMT characteristics in the primary tumour does not prove that EMT is even absolutely required for metastatic spread. In line with this, not all cells that have undergone EMT will successfully metastasize. Further studies suggest that EMT may be a temporal function of metastatic progression that may play a pivotal role and predict prognosis in earlier stages of CRC, as also described by our study groups, whereas successful spread may be dependent on various factors including transcription factors, miRNAs, and noncoding RNAs, epigenetic regulators, environmental factors, and multiple other signaling molecules [34].

We have previously assessed the relevance of consensus-molecular subgroups (CMS), grouping CRC according to their gene-signature in four different subtypes, and found that OS in CMS4 (defined by EMT and an activated tissue growth factor (TGF)-β pathway making this subgroup more chemo-resistant) favored FOLFIRI plus cetuximab over FOLFIRI plus bevacizumab. However, similar to this study and from a clinical standpoint, CMS classification appeared not to be of superior value with regard to patient selection and optimal treatment [14]. In summary, evaluation of EMT-related genes has prognostic relevance in non-metastatic CRC, however, after the tumour has spread, they do not appear to add further value.

There are also limitations of this study. The material that the gene-expression analysis in this investigation was based on, was predominantly derived from primary tumours, as liver tissue was not available in the majority of cases, due to irresectability. In this respect, EMT-related characteristics in the primary tumour may not have prognostic relevance after the primary tumour has spread, however, although it has been shown that CMS classification changes from primary tumour to metastases, it remains unclear whether the expression of EMT related genes changes within metastases, which would be an interesting research question for future studies [35]. We used a range of different methods to be able to capture different types of effects that genes could exhibit on OS or PFS (linear or non-linear effects, interactions, deviation from proportional hazards). The results indicate no prognostic effect of gene data, as defined by predictive performance measured by the IBS, however, given the usually small effect sizes associated with individual genes, the sample size for this study might have been too low in order to detect them, if present. Performance of GBT could probably be improved through additional tuning, however, given the results obtained from other learners, it is doubtful that performance of the KM could be improved upon.

Overall, EMT-related genes did not show prognostic relevance in stage IV CRC and are not of additional value compared to common parameters used regarding patient selection and clinical decision making. In this respect, further studies that investigate novel metrics that reflect tumour biology to predict therapeutic response and outcome in mCRC and that can supplement the currently limited decision-making tools are warranted.

## 5. Conclusions

We have assessed the prognostic relevance of EMT-related genes in stage IV CRC. The results of this study indicate that in an advanced stage, EMT-related characteristics may give insights into the underlying tumour biology and mechanisms that have preceded metastatic spread but do not add further value to the determination of prognosis compared to common parameters used regarding patient selection and clinical decision making.

## Figures and Tables

**Table 1:** Patient characteristics.

	Sub Category	Frequency	Percent (%)
Total valid records		350	100
Gender	Female	113	32.28
	Male	237	67.71
Type of treatment	Cetuximab	165	47.14
	Bevacizumab	185	52.86
Tumour location	Left	273	78.00
	Right	77	22.00
Solitary liver metastasis	Yes	116	33.14
	No	234	66.86
BRAF^V600E^	Wt	265	75.71
	Mut	20	5.71
	Not tested	65	18.57
Overall survival (OS)	Censored	37	10.57
	Dead	313	89.43
Progression-free survival (PFS)	Censored	21	6.00
	Progression or dead	329	94.00

**Table 2:** Benchmark experiments with respect to overall survival and progression-free survival. Aggregated predictive performance of five learners based on 25 values of the IBS. Lower values indicate better performance. Performance of the unregularised Cox model is omitted for the high-dimensional setting (data with genes). Performance of KM is identical between the different settings (clinical only vs. clinical + genetic).

Task	Learner	Mean (sd)	Median
OS (clinical only)	Kaplan-Meier	0.25 (0.005)	0.2496
	Cox	0.253 (0.006)	0.2522
	Regularised Cox	0.25 (0.005)	0.2496
	GBT	0.284 (0.019)	0.2837
	RSF	0.256 (0.007)	0.2550
OS (clinical + genetic)	Kaplan-Meier	0.250 (0.005)	0.2496
	Cox	—	—
	Regularised Cox	0.25 (0.005)	0.2496
	GBT	0.262 (0.009)	0.2619
	RSF	0.255 (0.007)	0.2551
PFS (clinical only)	Kaplan-Meier	0.251 (0.007)	0.2527
	Cox	0.257 (0.013)	0.2574
	Regularised Cox	0.251 (0.007)	0.2527
	GBT	0.276 (0.021)	0.2717
	RSF	0.256 (0.011)	0.2557
PFS (clinical + genetic)	Kaplan-Meier	0.251 (0.007)	0.2527
	Cox	—	—
	Regularised Cox	0.251 (0.007)	0.2527
	GBT	0.268 (0.013)	0.2697
	RSF	0.258 (0.009)	0.2585

## Data Availability

The data that support the findings of this study are available on request from the corresponding author. The data are not publicly available due to privacy or ethical restrictions.

## Appendix A

**Model**	**Hyperparameter**	**Searching Range**
Random Survival Forest	Minimum Node Size	(1, 50)
	Number of variables to possibly split at in each node	(0, 1)
	Fraction of observations to sample	(0.1, 1)
	Sample with replacement	(TRUE, FALSE)
	Splitting rule	(maxstat, logrank)
Cox (regularised)	Elastic net mixing parameter alpha; penalty parameter is trained internally via 10-fold CV	(0, 1)
Gradient Boosting Tree	Maximum depth of a tree	(1, 20)
	Subsample ratio of the training instances	(0.1, 1.0)
	Number of rounds	(10, 5000)
	Step size shrinkage	(0.01, 1.0)
	Subsample ratio of columns when constructing each tree	(0.01, 1.0)
	Minimum loss reduction required to make a further partition on a leaf node of the tree	(0.01, 3)
	The way new nodes are added to the tree	(Depth-wise, Loss-guide)

In order to assess predictive performance of different algorithms and data modalities (clinical and genetic), we set up a benchmark experiment with a nested cross-validation, 5 times repeated 5-fold CV in the outer loop (for performance evaluation) and a 3-fold CV in the inner loop for hyperparameter optimisation (HPO). Random searching mechanism is deployed during the optimisation, where the number of search iterations depends on the dimension of the search space. Concretely, the number of iterations is determined by the size of the search-space based on a linear growth strategy B = b + k * D in which B is the number of evaluations, D is the dimension of the search space, b is 25 and k is 60 in our experiments.
